# Supplementary material for: V-Set and Immunoglobulin Domain-Containing 1 (VSIG1), Predominantly Expressed in Testicular Germ Cells, Is Dispensable for Spermatogenesis and Male Fertility in Mice
Source: Animals (Basel). 2021 Apr 7;11(4):1037. doi: 10.3390/ani11041037 (PMC8067554; doi:10.3390/ani11041037)

## Slide 1
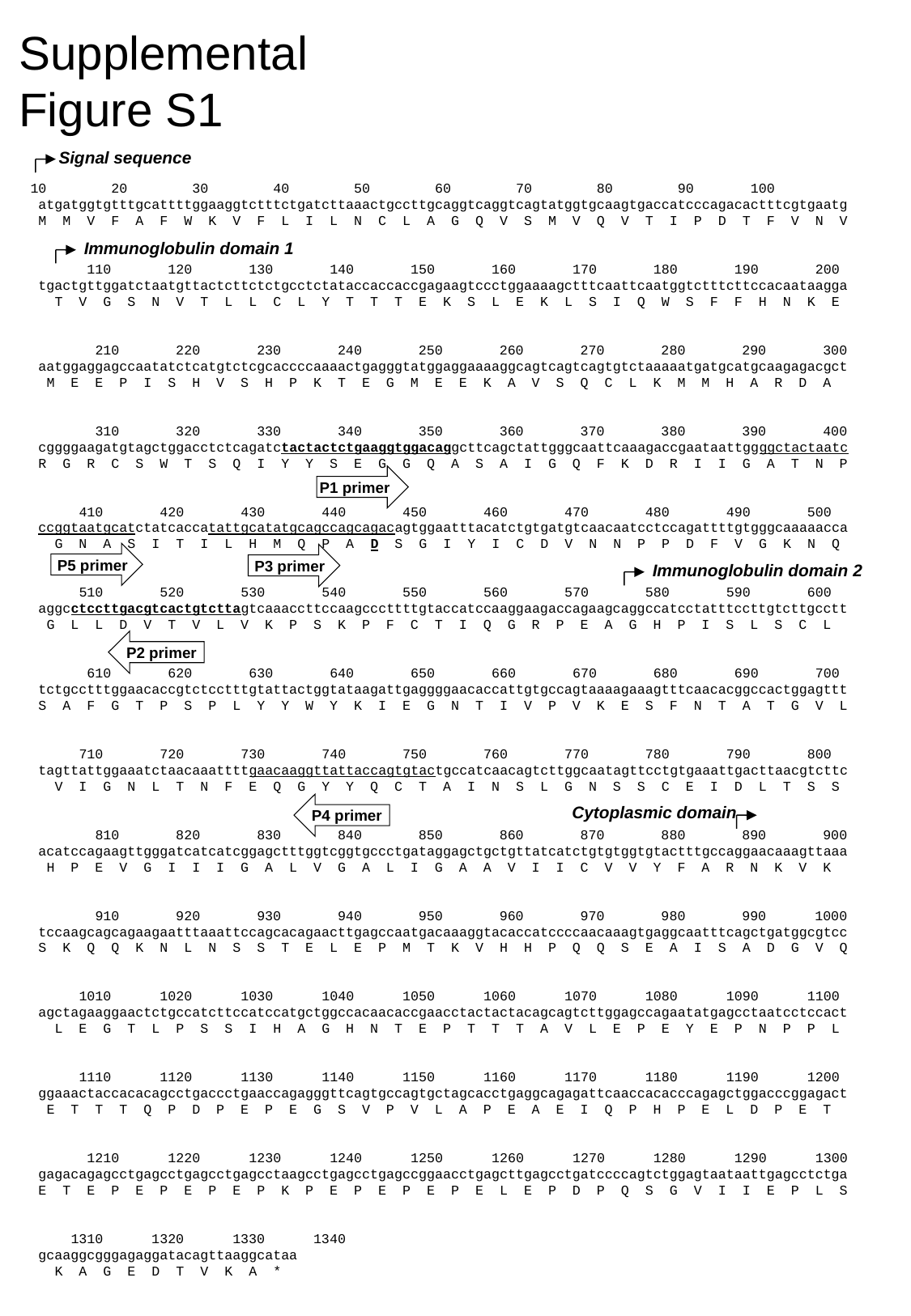

Supplemental Figure S1
Signal sequence
 10 20 30 40 50 60 70 80 90 100
 atgatggtgtttgcattttggaaggtctttctgatcttaaactgccttgcaggtcaggtcagtatggtgcaagtgaccatcccagacactttcgtgaatg
 M M V F A F W K V F L I L N C L A G Q V S M V Q V T I P D T F V N V
 110 120 130 140 150 160 170 180 190 200
 tgactgttggatctaatgttactcttctctgcctctataccaccaccgagaagtccctggaaaagctttcaattcaatggtctttcttccacaataagga
 T V G S N V T L L C L Y T T T E K S L E K L S I Q W S F F H N K E
 210 220 230 240 250 260 270 280 290 300
 aatggaggagccaatatctcatgtctcgcaccccaaaactgagggtatggaggaaaaggcagtcagtcagtgtctaaaaatgatgcatgcaagagacgct
 M E E P I S H V S H P K T E G M E E K A V S Q C L K M M H A R D A
 310 320 330 340 350 360 370 380 390 400
 cggggaagatgtagctggacctctcagatctactactctgaaggtggacaggcttcagctattgggcaattcaaagaccgaataattggggctactaatc
 R G R C S W T S Q I Y Y S E G G Q A S A I G Q F K D R I I G A T N P
 410 420 430 440 450 460 470 480 490 500
 ccggtaatgcatctatcaccatattgcatatgcagccagcagacagtggaatttacatctgtgatgtcaacaatcctccagattttgtgggcaaaaacca
 G N A S I T I L H M Q P A D S G I Y I C D V N N P P D F V G K N Q
 510 520 530 540 550 560 570 580 590 600
 aggcctccttgacgtcactgtcttagtcaaaccttccaagcccttttgtaccatccaaggaagaccagaagcaggccatcctatttccttgtcttgcctt
 G L L D V T V L V K P S K P F C T I Q G R P E A G H P I S L S C L
 610 620 630 640 650 660 670 680 690 700
 tctgcctttggaacaccgtctcctttgtattactggtataagattgaggggaacaccattgtgccagtaaaagaaagtttcaacacggccactggagttt
 S A F G T P S P L Y Y W Y K I E G N T I V P V K E S F N T A T G V L
 710 720 730 740 750 760 770 780 790 800
 tagttattggaaatctaacaaattttgaacaaggttattaccagtgtactgccatcaacagtcttggcaatagttcctgtgaaattgacttaacgtcttc
 V I G N L T N F E Q G Y Y Q C T A I N S L G N S S C E I D L T S S
 810 820 830 840 850 860 870 880 890 900
 acatccagaagttgggatcatcatcggagctttggtcggtgccctgataggagctgctgttatcatctgtgtggtgtactttgccaggaacaaagttaaa
 H P E V G I I I G A L V G A L I G A A V I I C V V Y F A R N K V K
 910 920 930 940 950 960 970 980 990 1000
 tccaagcagcagaagaatttaaattccagcacagaacttgagccaatgacaaaggtacaccatccccaacaaagtgaggcaatttcagctgatggcgtcc
 S K Q Q K N L N S S T E L E P M T K V H H P Q Q S E A I S A D G V Q
 1010 1020 1030 1040 1050 1060 1070 1080 1090 1100
 agctagaaggaactctgccatcttccatccatgctggccacaacaccgaacctactactacagcagtcttggagccagaatatgagcctaatcctccact
 L E G T L P S S I H A G H N T E P T T T A V L E P E Y E P N P P L
 1110 1120 1130 1140 1150 1160 1170 1180 1190 1200
 ggaaactaccacacagcctgaccctgaaccagagggttcagtgccagtgctagcacctgaggcagagattcaaccacacccagagctggacccggagact
 E T T T Q P D P E P E G S V P V L A P E A E I Q P H P E L D P E T
 1210 1220 1230 1240 1250 1260 1270 1280 1290 1300
 gagacagagcctgagcctgagcctgagcctaagcctgagcctgagccggaacctgagcttgagcctgatccccagtctggagtaataattgagcctctga
 E T E P E P E P E P K P E P E P E P E L E P D P Q S G V I I E P L S
 1310 1320 1330 1340
 gcaaggcgggagaggatacagttaaggcataa
 K A G E D T V K A *
 Immunoglobulin domain 1
P1 primer
P5 primer
P3 primer
 Immunoglobulin domain 2
P2 primer
P4 primer
 Cytoplasmic domain

## Slide 2
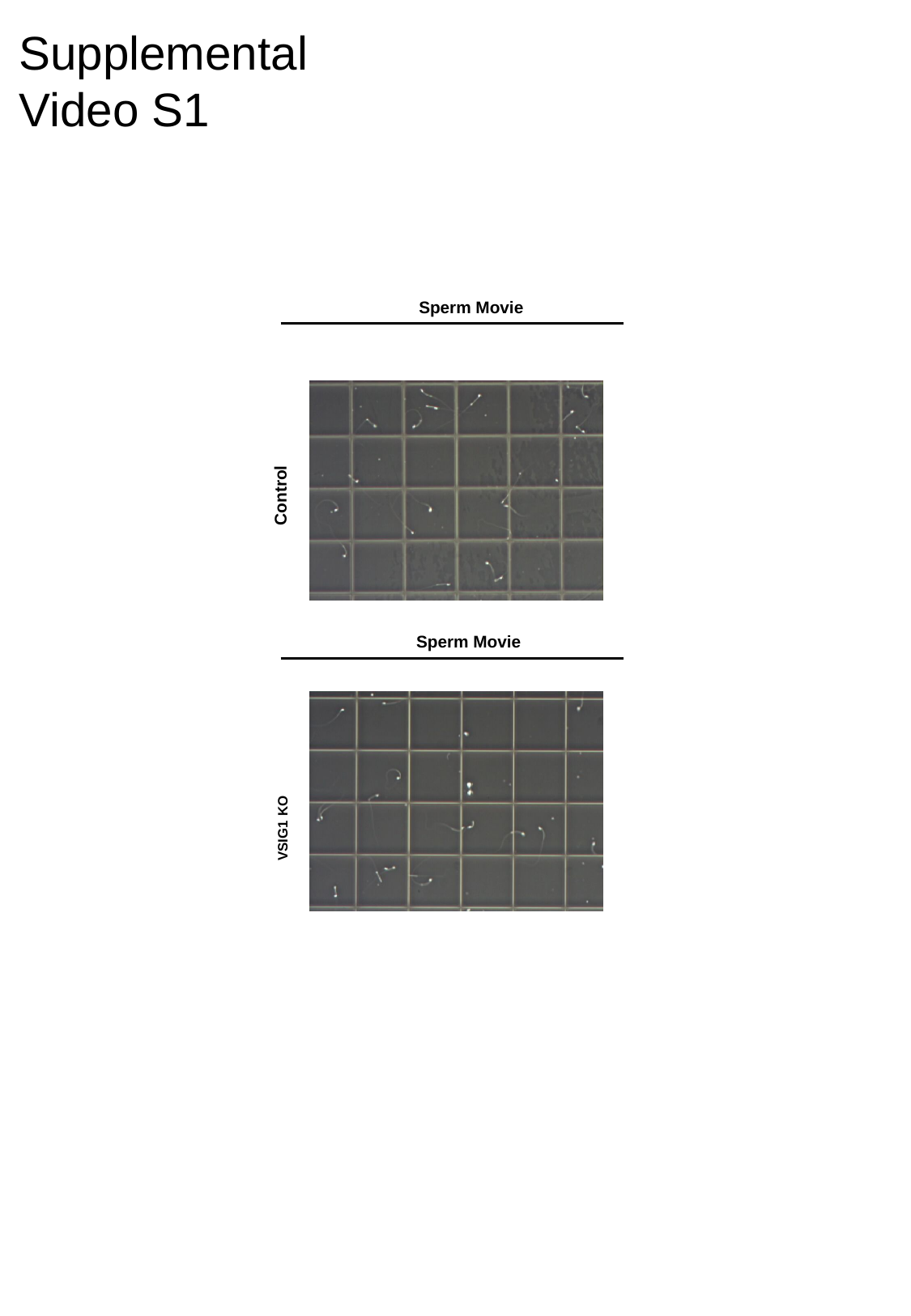

Supplemental
Video S1
Sperm Movie
Sperm Movie
Control
VSIG1 KO

## Slide 3
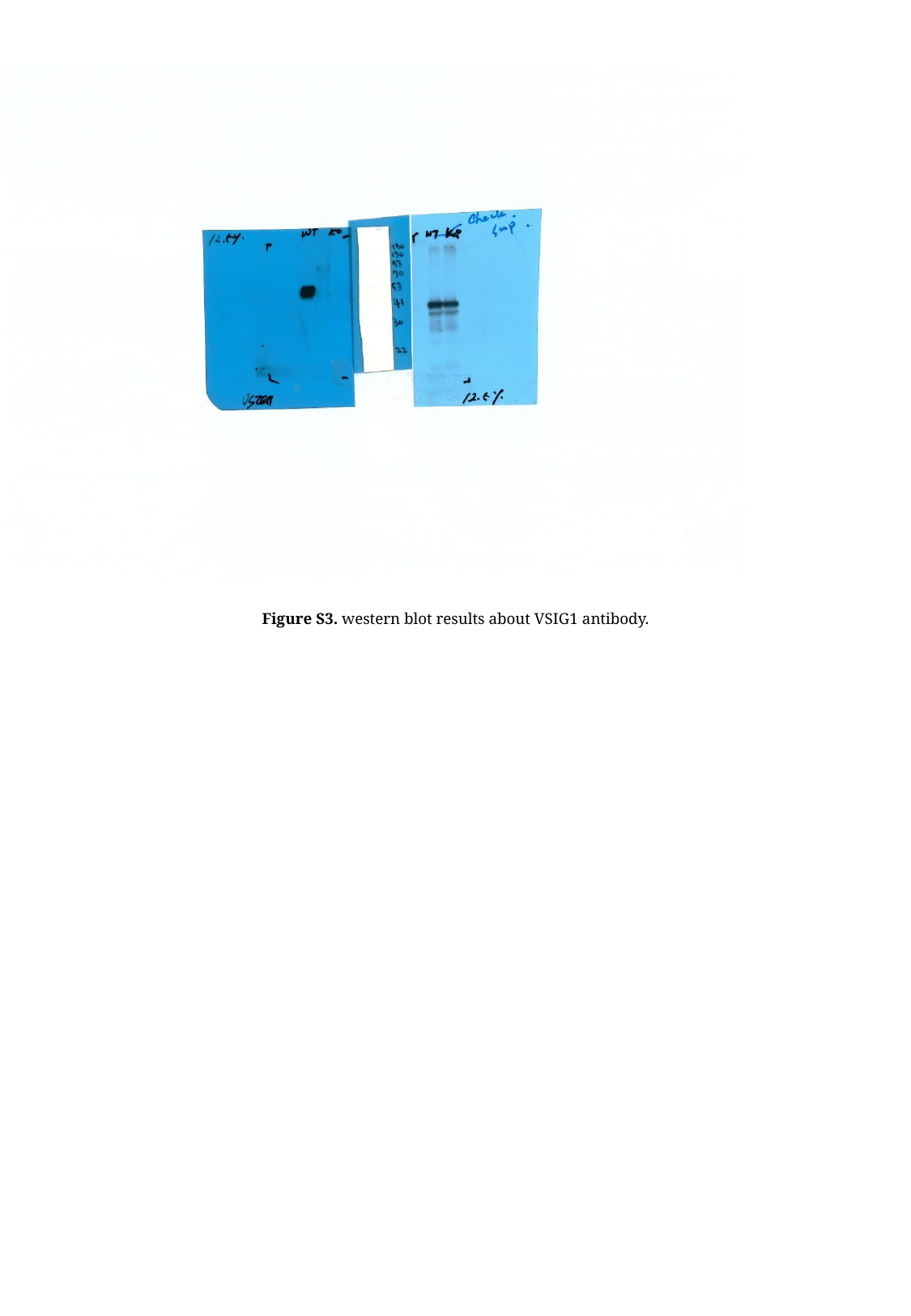

Figure S3. western blot results about VSIG1 antibody.

## Slide 4
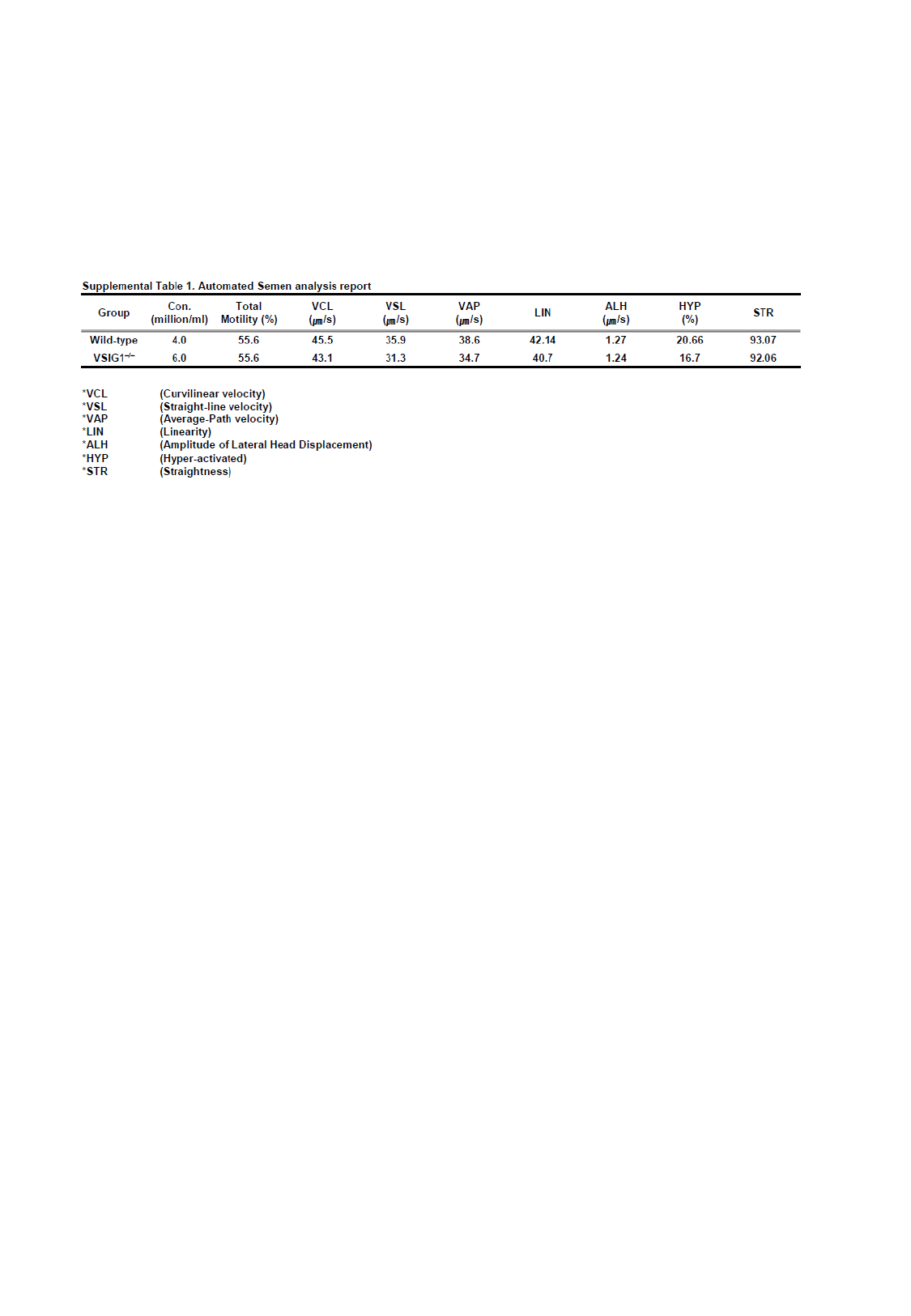

Supplement: Supplementary file 1 [file animals-11-01037-s001.zip › animals-1126569-supplementary.pptx]
